# The One Discredited Material

**Published:** 1894-06

**Authors:** 


### Editorial.

### THE ONE DISCREDITED MATERIAL.

IN the early history of dental practice materials for filling teeth were limited to the metals or to the resins, and where a better class of work was demanded there was but one material to use,—gold. It is probable that lead was first used, but as the distinction between tin and lead was not made until the fourth century, it is reasonable to conclude that where lead was stated, tin really was meant. The use, therefore, of the latter as a filling-material is lost in the mists of antiquity.

Of one thing, however, we may feel assured, that at no period has this metal been regarded with favor by the majority of practitioners; indeed, it has remained the contemned material from the beginning by the many and by the few treasured as next to gold, the most valuable filling-material in the list of metals.

The history of tin, as far as we have it, is one of the most curious and interesting of any part of dentistry. At the period when materials were limited, it found but little favor. It was the stuff for the poor, and it has been from that time to the present, in the opinion of the majority, unfit for the good teeth, but able to perform a fairly reasonable service for frail structures. It has been the "ugly duckling" of the dental profession, and if it becomes in the end the graceful "swan," it will have been through tribulations unspeakable.

Harris says of it, in his fourth edition (1850), "Properly prepared it will sometimes effectually preserve the organ, but if the fluids of the mouth are very much vitiated, or should they at any time afterwards become so, it soon oxidizes and turns black, and then, instead of preventing, it rather promotes a recurrence of the disease."

Taft (1877) writes, "Some cases seemingly favorable to its use are found, on examination, to be otherwise; and in almost any mouth in which there is a large proportion of mucus secreted, it cannot be depended upon for permanency."

From these two quotations—and more could be given—it is seen that this metal has occupied a discredited position; and, while at times used, it was always under an implied protest.

The manufacture of tin into leaves, similar to gold, while accomplished with apparent ease, was not attended with equally good results. Gold, in the form of non-cohesive and eventually cohesive, was supplied to the dentist in various forms, but tin received but little attention. The renaissance of this material, if we may judge by the discussions in our various societies, is at hand. This was begun at the World's Columbian Congress, and supplemented by the able presentation of Professor Darby before the Second District Dental Society of New York. This exhibit of what could be done with tin under modern processes of manufacture and proper manipulation has seemingly aroused much interest, with a prospect that this much-abused metal may yet take its rightful place in the royal line of materials.

In view of this probable result, it may not be inappropriate to review some of the objections urged against it. The following quotation is taken from the *Dental Cosmos* of May, 1894, in an article by R. C. Brewster, M.D., D.D.S., on tin-foil, in which the writer almost pathetically gives his experience in its use, for he says, "To this end I bought book after book of tin-foil, that which was freshly beaten and that which was old, and did my level best, using serrated points, broken points, and smooth points, with hand-pressure and the various kinds of mallets, although a combination of hand-pressure and hand-mallet, with finely serrated points, worked the best, . . . and yet," he says further on, "I could never condense such a filling to make it a solid homogeneous mass."

We have no doubt of the ability of the writer, and it would seem that he had exhausted every conceivable means and methods in his efforts to use this material. It cannot, therefore, be justly charged to lack of energy. It has been the experience of every dental educator that in any given class of students there will be a few unable to manipulate gold-foil, and all efforts, with the best instruction and the most satisfactory form of instruments, fail of any result. With some this is a natural defect never overcome, with others it is simply that they have not been able to strike the right cord and are out of harmony with their work. The time comes when suddenly a new inspiration falls upon these, and they advance in the use of gold beyond their fellows. It is a well-known fact that some of our best operators have gone through this experience. It is, therefore, assumed that our friend needs a new inspiration.

The objections that it oxidizes, turns black, is easily penetrated by an excavator, disintegrates, will not bear the force of mastication, its preservative properties overrated, and, finally, it has no cohesive property, if true, should condemn it.

Now, what are the facts? If properly packed in the cavity, it does *not* turn black, but simply slightly changes its color, in many cases not more than gold, and rarely equalling in this respect the best preparations of amalgam. If well inserted, it will be found difficult to penetrate, but in this respect it is not equal to gold. It will not disintegrate in proper positions more rapidly than gold, if equally well inserted. It will bear the force of mastication with remarkable results. This has been demonstrated in the experience of the writer in many cases of twenty-five years' wear on masticating surfaces. Its preservative properties have been so severely tested, and have proved so valuable in connection with its poor conducting qualities, that this constitutes an important reason for its more extended use. The objection that it is non-cohesive cannot be sustained.

All metals possess the property of cohesion under proper conditions; tin is no exception.

The use of tin was limited because of inferior manufacture. Good tin, in the form of foil, must be throughout bright upon the surface. Where it is spotted, streaked, or entirely coated with oxidation, it should be rejected.

The writer spent much time twenty-five years ago in efforts to improve the quality of tin then in the market. It was found that by shaving pure tin the pieces were perfectly cohesive, uniting without pressure, in the same manner and with the same force as cohesive gold. This property proved, however, evanescent, the metal losing this perfect cohesive property by oxidation, rendering it of no practical value. Dr. Darby's original investigations reintroduced this quality at the meeting named, creating much interest. The manufacture of tin-foil has greatly improved of latter years, and it is now sufficiently cohesive for all practical purposes.

A mistake has probably been made by operators in using short serrated instruments. The old-style long and sharp serrations are to be preferred.

The preparation of the tin for use is of some importance. It should not be folded on itself, but cut in strips, packed by hand, and consolidated by the mallet.

One quality tin possesses, so important that it is surprising it has not been noticed,—that of working almost as well under water as when dry. No other metal or plastic filling will give equally as good results thus treated. This is a most desirable quality, especially for use in children's teeth, where the difficulties from moisture are almost insurmountable. All that is required is to keep the filling well syringed as the operation proceeds. So well assured is the writer of the value of this and the lasting qualities of fillings of tin placed in under water, that in children no attention is paid in practice to dryness, and rubber dam and napkins will be found unnecessary. Equally good results can be obtained in mature mouths.

Tin, therefore, in the sense of use, is a royal metal to the dentist, and while the supply-houses show by their reports that it is a discredited material, we are very sure that there is a large minority who would not part with it for their own comfort and the good of their patients.

